# Emodin Sensitizes Hepatocellular Carcinoma Cells to the Anti-Cancer Effect of Sorafenib through Suppression of Cholesterol Metabolism

**DOI:** 10.3390/ijms19103127

**Published:** 2018-10-12

**Authors:** Young-Seon Kim, Yoon-Mi Lee, Taek-In Oh, Dong Hoon Shin, Geon-Hee Kim, Sang-Yeon Kan, Hyeji Kang, Ji Hyung Kim, Byeong Mo Kim, Woo Jong Yim, Ji-Hong Lim

**Affiliations:** 1 Department of Biomedical Chemistry, College of Biomedical & Health Science, Konkuk University, Chungju 27478, Chungbuk, Korea; yskim0801@kku.ac.kr (Y.-S.K.); dk1050@kku.ac.kr (T.-I.O.); rlarjsgml4@kku.ac.kr (G.-H.K.); hsb6477@kku.ac.kr (S.-Y.K.); kkang@kku.ac.kr (H.K.); 2 Department of Food Bioscience, College of Biomedical & Health Science, Konkuk University, Chungju 27478, Chungbuk, Korea; yoonmilee@kku.ac.kr; 3 Research Institute, National Cancer Center, Department of Cancer Biomedical Science, National Cancer Center Graduate School of Cancer Science and Policy, Goyang 10408, Korea; dhshin@ncc.re.kr; 4 College of Life Sciences and Biotechnology, Korea University, Seoul 02841, Korea; jay_kim@korea.ac.kr; 5 Severance Integrative Research Institute for Cerebral & Cardiovascular Diseases (SIRIC), Yonsei University College of Medicine, Seodaemun-gu, Seoul 03722, Korea; bkim2@yuhs.ac; 6 Jung-Ang Microbe Research Institute (JM), 398, Jikji-daero, Heungdeok-gu, Cheongju 28576, Chungbuk, Korea; ywj0808@naver.com; 7 Diabetes and Bio-Research Center, Konkuk University, Chungju 27478, Chungbuk, Korea

**Keywords:** emodin, sorafenib, combination, cholesterol, hepatocellular carcinoma

## Abstract

Reduced therapeutic efficacy of sorafenib, a first-generation multikinase inhibitor, is often observed during the treatment of advanced hepatocellular carcinoma (HCC). Emodin is an active component of Chinese herbs, and is effective against leukemia, lung cancer, colon cancer, pancreatic cancer, and HCC; however, the sensitizing effect of emodin on sorafenib-based HCC therapy has not been evaluated. Here, we demonstrate that emodin significantly improved the anti-cancer effect of sorafenib in HCC cells, such as HepG2, Hep3B, Huh7, SK-HEP-1, and PLC/PRF5. Mechanistically, emodin inhibits sterol regulatory element-binding protein-2 (SREBP-2) transcriptional activity, which suppresses cholesterol biosynthesis and oncogenic protein kinase B (AKT) signaling. Additionally, attenuated cholesterol synthesis and oncogenic AKT signaling inactivated signal transducer and activator of transcription 3 (STAT3), an oncogenic transcription factor. Furthermore, emodin synergistically increased cell cycle arrest in the G1 phase and apoptotic cells in the presence of sorafenib. Animal models xenografted with HepG2 or SK-HEP-1 cells also showed that the combination of emodin and sorafenib was sufficient to inhibit tumor growth. Overall, these results suggested that the combination of emodin and sorafenib may offer a potential therapy for patients with advanced HCC.

## 1. Introduction

Hepatocellular carcinoma (HCC), which is closely associated with chronic liver diseases, particularly viral hepatitis and metabolic steatohepatitis, is the third most common cause of cancer-related deaths worldwide [1,2]. Sorafenib, as a small-molecule inhibitor that targets multiple types of kinases required for tumor growth, angiogenesis, and metastasis, has been used as the only standard treatment available for advanced HCC [1]. However, a previous clinical study in patients with advanced HCC showed that the median overall survival was improved by only a few months in the sorafenib group compared with that in the placebo group, which implied that there were no significant differences between the groups [3]. Thus, drugs that can sensitize the anti-cancer efficacy of sorafenib would confer beneficial effects on HCC treatment. 

Emodin (3-methyl-1,6,8-trihydroxyanthraquinone) is an active compound predominantly found in the rhizome of *Rheum palmatum* L. [4]. Many types of biologically active compounds that are used widely for cancer treatment, such as doxorubicin and paclitaxel, are derived from nature. Similarly, recent studies have shown that emodin also has anti-cancer effects in different types of cancers, including leukemia, lung cancer, colon cancer, gallbladder cancer, pancreatic cancer, breast cancer, and HCC [5,6]. Mechanistically, emodin suppresses cell growth and proliferation through the attenuation of oncogenic growth signaling, such as Wnt/β-catenin, HER-2 tyrosine kinase, mitogen-activated protein kinase (MAPK), phosphatidylinositol 3-kinase (PI3K), and protein kinase B (AKT), which leads to apoptosis in several cancer cell types [7,8,9]. Interestingly, several recent studies have shown that emodin could synergistically improve the anti-cancer efficacy of conventional chemotherapeutic drugs, such as gemcitabine, paclitaxel, cisplatin, and etoposide, in pancreatic cancer, malignant melanoma, and HER-2/neu-overexpressing lung cancer [10,11,12,13]. Nevertheless, the ability of emodin to sensitize cells to the anti-cancer efficacy of molecular targeted cancer therapies, such as sorafenib, has not been investigated in HCC. Thus, we have investigated whether emodin exerted beneficial effects to improve the anti-cancer efficacy of sorafenib in HCC therapy. 

Anabolic metabolism, including cholesterol biosynthesis, which is also called cholesterogenesis, is considered to be a hallmark of cancer [14]. Evidence has emerged to indicate that the biosynthesis of fatty acids and cholesterol is essential for the development and progression of a wide variety of tumors, owing to their critical nature as building blocks for membrane components [15]. In addition, increased intracellular cholesterol levels were closely associated with the subsequent alterations of oncogenic growth signaling and motility in cancer cells [14]. Intracellular cholesterol levels are mainly controlled by sterol regulatory element-binding protein-2 (SREBP-2), a transcription factor that regulates genes encoding a variety of enzymes required for cholesterogenesis [16]. Mechanistically, SREBP-2 transcriptionally activates the expression of cholesterogenic genes in cholesterol-depleted conditions, such as hydroxymethylglutaryl (HMG)-CoA synthase 1 (HMGCS1), HMG-CoA reductase (HMGCR), farnesyl diphosphate synthase (FDPS), and mevalonate diphosphate decarboxylase (MVD) [16]. Although the cholesterogenic pathway is considered to be a promising pharmaceutical target for cancer treatment, the ability to sensitize HCC cells to the effect of cholesterol-lowering drugs and improve the anti-cancer effect has been poorly studied. 

We hypothesized that the combination of emodin and sorafenib would lead to synergistic anti-cancer efficacy of HCC therapy. In the present study, we have shown that the combination of emodin and sorafenib functioned synergistically to increase cell cycle arrest and the proportion of apoptotic cells, which was consistent with the observed decrease in cell viability, through the suppression of oncogenic AKT signaling and activation of signal transducer and activator of transcription 3 (STAT3) in HCC cells. We also found that the cholesterol-lowering effect of emodin, mediated through the suppression of SREBP-2 transcriptional activity and its target gene expression, was involved in the combined anti-cancer efficacy with sorafenib. Moreover, we suggested that the combination treatment of both emodin and sorafenib would act synergistically to produce a more effective anti-cancer effect in HepG2 and SK-HEP-1 cell-transplanted xenograft models than monotherapy with sorafenib. Overall, our results have demonstrated that the combination of emodin and sorafenib may be a promising strategy to achieve improvements in the therapeutic efficacy of sorafenib in patients with advanced HCC. 

## 2. Results

### 2.1. Synergistic Anti-Cancer Effect of Combination of Emodin and Sorafenib in HCC Cells

**Figure 1:**
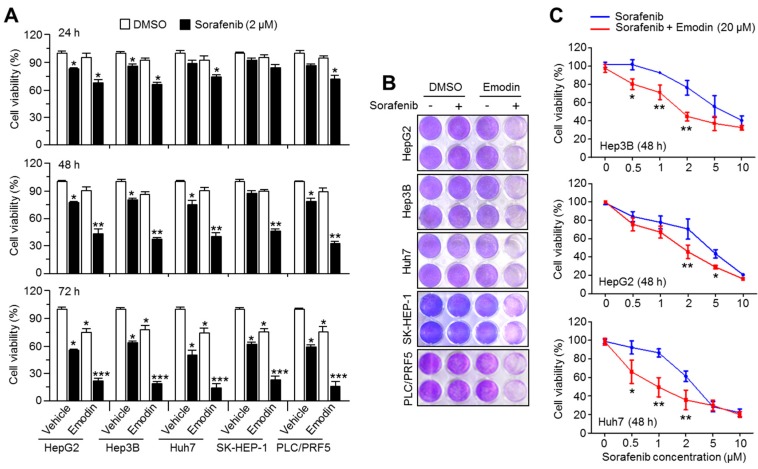
Emodin sensitizes hepatocellular carcinoma (HCC) cells to the anti-cancer effect of sorafenib. (**A**) Cell viability analysis after emodin, sorafenib, or the combination treatment of both drugs in five HCC cell lines. The cells were incubated with emodin (20 μM), sorafenib (2 μM), or the combination (20 μM emodin and 2 μM sorafenib) for 24, 48, or 72 h, as indicated. The values represent the mean ± SEM of three independent experiments performed in triplicate; * *p* < 0.05, ** *p* < 0.01, and *** *p* < 0.001. (**B**) Images of crystal violet staining. (**C**) The cell viability after different concentrations of sorafenib (0.5, 1, 2, 5, and 10 μM) with 20 μM emodin in Hep3B, HepG2, and Huh7 cells. The values represent the mean ± SEM of three independent experiments performed in triplicate; * *p* < 0.05 and ** *p* < 0.01.

Emodin, a bioactive compound found in many species of plants, including rhubarb and buckthorn, has been shown to have anti-cancer effects in multiple types of cancer; however, its ability to sensitize HCC cells to the anti-cancer efficacy on sorafenib therapy has been not elucidated. Here, we first evaluated the sensitizing efficacy of emodin on the growth inhibition of HCC cells induced by 2 μM of sorafenib. The treatment with 20 μM of emodin strongly enhanced the suppressive effect of sorafenib on HCC cell growth in a time-dependent manner (Figure 1A,B). To elucidate whether emodin was sufficient to enhance the anti-cancer activity of lower concentrations of sorafenib, the cell viability was measured after the treatment with 20 μM emodin and different concentrations of sorafenib. Unexpectedly, the sensitizing anti-cancer effect of 20 μM emodin was observed to occur with 0.5 μM and 1 μM sorafenib treatment in Hep3B and Huh7 cells (Figure 1C). In addition, Figure 1C also shows that when HCC cells are exposed to higher concentration of sorafenib than 2 μM with 20 μM of emodin, sensitizing anti-cancer effects induced by combination of both drugs were not observed (Figure 1C). These results suggest that emodin could increase the anti-cancer activity upon lower concentration of sorafenib. 

### 2.2. Emodin Did Not Sensitize HCC to the Anti-Cancer Activity of Doxorubicin or 5-Fluorouracil

**Figure 2:**
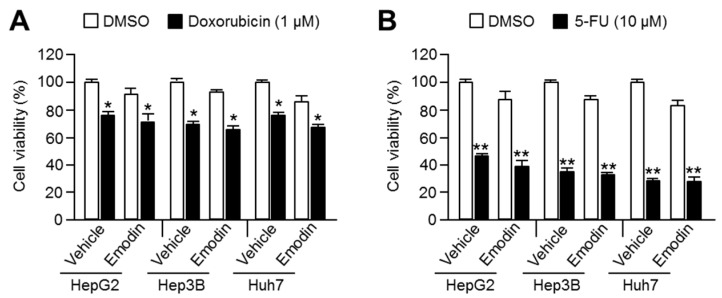
Emodin did not sensitize HCC to the anti-cancer effect of doxorubicin or 5-fluorouracil. (**A**) The viability of three HCC cell lines after treatment with of emodin and doxorubicin. (**B**) The combinatory anti-cancer effect of emodin and 5-fluorouracil in the HCC cell lines. Cells were treated with the indicated drugs (1 μM doxorubicin or 10 μM 5-fluorouracil) for 48 h in the absence or presence of 20 μM emodin. The values are presented as the mean ± SEM of three independent experiments performed in duplicate; * *p* < 0.05 and ** *p* < 0.01.

Doxorubicin and 5-fluorouracil are two of the first-line chemotherapeutic drugs used for HCC treatment [1]. To investigate the specificity of emodin to sensitize HCC cells to the anti-cancer effects of sorafenib, the efficiency of the combination of emodin was tested with these standard chemotherapeutic drugs. However, emodin did not exert synergistic anti-cancer effects when combined with doxorubicin (Figure 2A) or 5-fluorouracil (Figure 2B) treatment.

### 2.3. Combination Therapy of Emodin and Sorafenib Caused Cell Cycle Arrest and Apoptosis in HCC Cells

**Figure 3:**
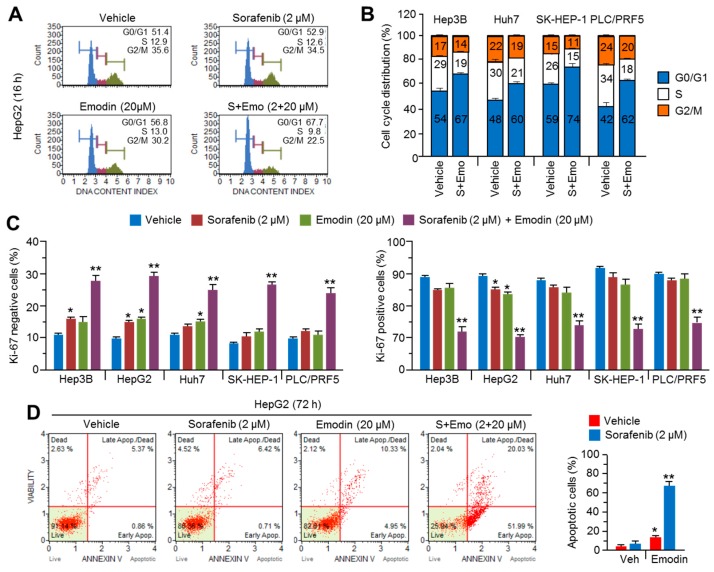
The combination therapy of emodin and sorafenib causes cell cycle arrest and apoptosis in HCC cells. (**A**) The cell cycle analysis after treatment with the combination of emodin and sorafenib in HepG2 cells. (**B**) The cell cycle analysis after treatment with the combination of emodin and sorafenib in Hep3B, Huh7, SK-HEP-1, and PLC/PRF5 cells. The cells were incubated with emodin (20 μM), sorafenib (2 μM), or the combination (20 μM emodin and 2 μM sorafenib) for 16 h. The values represent the mean ± SEM of three independent experiments performed in duplicate. (**C**) Cell proliferation after treatment with the combination of emodin and sorafenib in five HCC cell lines. The cells were treated with the indicated drugs for 24 h prior to analysis. The values represent the mean ± SEM of three independent experiments performed in duplicate. (**D**) The induction of apoptosis after treatment with the combination of emodin and sorafenib in HepG2 cells. The cells were incubated with emodin and sorafenib for 72 h. The values represent the mean ± SEM of three independent experiments performed in duplicate; * *p* < 0.05 and ** *p* < 0.01.

As sorafenib suppressed tumor growth through cell cycle arrest and apoptosis [17], we investigated whether emodin sensitized HCC cells to the antiproliferative and pro-apoptotic effects of sorafenib. Cell cycle arrest of the G1 phase of approximately 15% was observed after treatment with the combination of emodin and sorafenib; however, treatment with a single compound did not alter the cell cycle distribution in HepG2 cells (Figure 3A). The combination of emodin and sorafenib consistently induced G1 phase arrest in four different HCC cell lines (Figure 3B). When cell cycle arrest in the G1 phase occurs, the cells cease DNA replication and proliferation to maintain genomic stability [18]. The combination of emodin and sorafenib significantly increased the Ki67-negative cell population but decreased the Ki67-positive cell population, which indicated that the combination of emodin and sorafenib suppressed cell proliferation (Figure 3C). As prolonged cell cycle arrest and suppressed proliferation led to apoptosis [18], the pro-apoptotic efficacy of combination treatments of emodin and sorafenib was evaluated. Treatment with 2 μM sorafenib or 20 μM emodin resulted in a slight (approximately 8%) increase in apoptosis; however, the combination therapy with both drugs strongly increased the apoptotic cell population, by approximately 55% (Figure 3D). These results indicated that emodin synergistically enhanced the anti-cancer efficacy of sorafenib through effects on the cell cycle progression and apoptosis.

### 2.4. Emodin Suppressed Cholesterogenic Gene Expression and Intracellular Cholesterol Levels through the Attenuation of SREBP-2 in HCC Cells

**Figure 4:**
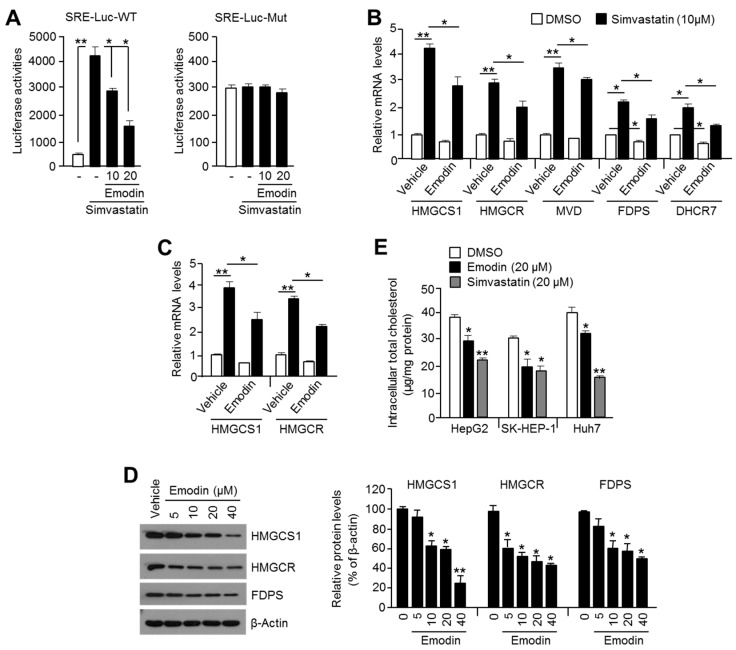
Emodin suppresses SREBP-2 transcriptional activity and decreases intracellular cholesterol. (**A**) The steroid-responsive element (SRE) containing luciferase vectors (SRE–Luc–WT or SRE–Luc–Mut) were transiently transfected into SK-HEP-1 cells. Transfected cells were incubated with 10 or 20 μM emodin in the absence or presence of 10 μM simvastatin for 24 h. The values represent the mean ± SD of three independent experiments performed in duplicate; * *p* < 0.05 and ** *p* < 0.01. (**B**) The suppressive effect of emodin on simvastatin-induced cholesterogenic gene expression. SK-HEP-1 cells were incubated with 20 μM emodin in the absence or presence of 10 μM simvastatin for 24 h. The gene expression was measured by quantitative real-time PCR, and relative mRNA expression was normalized to the expression of 36B4. The values represent the mean ± SEM of three independent experiments performed in triplicate; * *p* < 0.05 and ** *p* < 0.01. (**C**) The suppressive effect of emodin on simvastatin-induced cholesterogenic gene expression in HepG2 cells. (**D**) The inhibitory effect of emodin on cholesterogenic enzyme expression in SK-HEP-1 cells. The cells were incubated with 5, 10, 20, or 40 μM emodin for 24 h, and protein expression was analyzed by Western blotting, as described in the Materials and Methods. Relative protein expression levels are shown. The values are presented as the mean ± SD of three independent experiments performed; * *p* < 0.05 and ** *p* < 0.01. (**E**) Intracellular cholesterol level after emodin treatment. Three HCC cell lines were cultured for 48 h in the absence or presence of 20 μM emodin or 20 μM simvastatin. The values are presented as the mean ± SD of three independent experiments performed in duplicate; * *p* < 0.05 and ** *p* < 0.01.

Previously, the cholesterol-lowering effect of emodin through the suppression of cholesterogenic gene expression, such as 3-hydroxy-3-methylglutaryl-CoA synthase 1 (HMGCS1), 3-hydroxy-3-methylglutaryl-CoA reductase (HMGCR), and farnesyl pyrophosphate synthase (FDPS), has been reported in a high fat diet-induced obese mice model [19]. Cholesterogenic gene expression is tightly regulated through SREBP-2 translocation into the nucleus and transcriptional activation upon decreased intracellular cholesterol [16]. Because cholesterogenesis is an essential anabolic process to provide lipid building block components for the cellular membrane during cancer cell growth [14], we observed whether emodin also suppresses cholesterogenic gene expression and cholesterogenesis in HCC cells. Simvastatin-induced luciferase activity of steroid response element (SRE) was attenuated by emodin in a dose-dependent manner in wild-type (SRE–Luc–WT) cells, but not in mutant (SRE–Luc–Mut) luciferase vector-expressing cells, which suggested that emodin may inhibit SREBP-2 transcriptional activity (Figure 4A). Similarly, the induction of SREBP-2 target cholesterogenic genes, such as HMGCS1, HMGCR, mevalonate diphosphate decarboxylase (MVD), FDPS, and 7-dehydrocholesterol reductase (DHCR7) expression, by simvastatin was significantly decreased by 20 μM emodin in SK-HEP-1 (Figure 4B) and HepG2 (Figure 4C) cells. In addition, the cholesterogenic proteins, HMGCS1, HMGCR, and FDPS, were decreased by emodin in SK-HEP-1 cells in a dose-dependent manner (Figure 4D). We subsequently observed that emodin as well as simvastatin lowered the total intracellular cholesterol content in HepG2, SK-HEP-1, and Huh7 cells (Figure 4E). These results revealed that emodin attenuated cholesterol biosynthesis through the suppression of SREBP-2 transcriptional activity in HCC cells.

### 2.5. Combination of Emodin and Sorafenib Synergistically Suppresses Cholesterogenic Genes Expression and Causes Cell Death in HCC Cells

**Figure 5:**
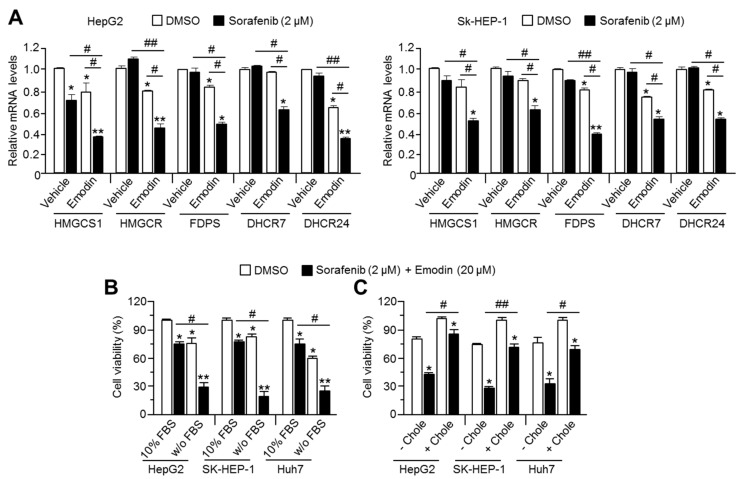
Suppression of cholesterol metabolism is required to sensitize HCC cells to the anti-cancer effect of sorafenib. (**A**) Cholesterogenic gene expression after the combination treatment of emodin and sorafenib in HepG2 and SK-HEP-1 cell lines. The cells were incubated with emodin (20 μM), sorafenib (2 μM), or their combination (20 μM emodin and 2 μM sorafenib) for 24 h. The values represent the mean ± SEM of three independent experiments performed in duplicate; * *p* < 0.05, ** *p* < 0.01, # *p* < 0.05, and ## *p* < 0.01. (**B**) The cell viability after the combination treatment of emodin and sorafenib in the absence or presence of 10% fetal bovine serum (FBS). Three cell lines were incubated with 10% FBS or FBS-free serum for 1 h prior to the combination treatment of emodin and sorafenib. Subsequently, the cells were incubated for 24 h with the indicated treatments. The values are presented as the mean ± SEM of three independent experiments performed in triplicate; * *p* < 0.05, ** *p* < 0.01, and # *p* < 0.05. (**C**) The cell viability after the combination treatment of emodin and sorafenib in the absence or presence of water-soluble cholesterol (0.5 mM). Three cell lines were incubated with or without 0.5 mM water-soluble cholesterol for 1 h prior to the combination treatment with emodin and sorafenib. Subsequently, the cells were incubated for a further 48 h. The values represent the mean ± SEM of three independent experiments performed in triplicate; * *p* < 0.05, ** *p* < 0.01, # *p* < 0.05, and ## *p* < 0.01.

To evaluate the effect of the combination of emodin and sorafenib on cholesterogenic gene expression, we analyzed HMGCS1, HMGCR, FDPS, DHCR7, and DHCR24 after treatment of HCC cells with emodin and sorafenib. We observed a synergistic effect of the combination treatment compared with the single drug treatment on cholesterogenic gene expression in HepG2 and SK-HEP-1 (Figure 5A). As the intracellular cholesterol levels are balanced by biosynthesis and supplementation from blood cholesterol [16], the combinatory anti-cancer effect of emodin and sorafenib was tested in the absence or presence of fetal bovine serum (FBS), which contains large amounts of cholesterol. The viability of HepG2, SK-HEP-1, and Huh7 cells was rapidly decreased by combination of emodin and sorafenib in the absence of FBS (Figure 5B). Moreover, we found that supplementation of water-soluble cholesterol significantly prevented the rapid cell death caused by the combination therapy of emodin and sorafenib in HCC cells (Figure 5C). These results demonstrated that the suppression of cholesterogenesis is important for the synergistic anti-cancer efficacy of the combination therapy of emodin and sorafenib.

### 2.6. Emodin Suppressed Oncogenic AKT Signaling Caused by Intracellular Cholesterol Depletion in HCC Cells

**Figure 6:**
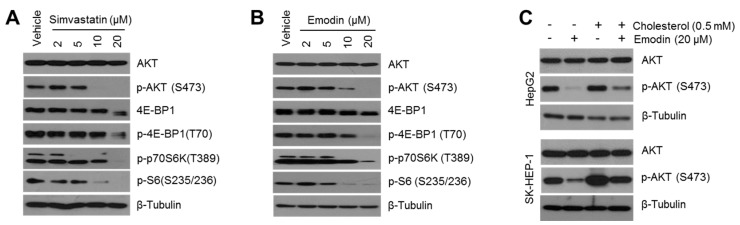
Decreased intracellular cholesterol level by emodin causes the suppression of the oncogenic protein kinase B (AKT) signaling pathway. (**A**) Simvastatin suppressed the AKT signaling pathways. SK-HEP-1 cells were incubated with different concentrations of simvastatin (2, 5, 10, and 20 μM) for 12 h. (**B**) Emodin suppressed the phosphorylation of AKT and its target substrates. SK-HEP-1 cells were incubated with emodin (2, 5, 10, and 20 μM) for 12 h. (**C**) The supplementation of water-soluble cholesterol rescued the decreased phosphorylation of AKT caused by emodin. SK-HEP-1 and HepG2 cells were incubated in the presence or absence of 0.5 mM cholesterol for 1 h prior to emodin treatment. Subsequently, the cells were incubated for a further 12 h, and protein expression was measured by Western blotting.

Evidence has emerged to indicate that increased lipids and cholesterol, which are correlated with elevated oncogenic growth signaling, such as PI3K-AKT and MAPK signaling in cancer cells, are now considered a hallmark of cancer aggressiveness [14,20]. Indeed, many reports have shown that cholesterol-lowering drugs, such as atorvastatin, lovastatin, and simvastatin, inhibit cancer cell growth and invasiveness via the suppression of oncogenic signaling in HCC, melanoma, glioma, and ovarian cancer [21,22,23,24]. On this basis, the inhibitory effect of emodin on oncogenic growth signaling in HCC cells was evaluated. To confirm whether the cholesterol-lowering drug was sufficient to suppress oncogenic signaling, the phosphorylation of AKT signaling was determined after simvastatin treatment in HCC cells. AKT signaling was decreased in simvastatin-treated SK-HEP-1 cells in a dose-dependent manner (Figure 6A), and as shown in Figure 6B, similar to simvastatin, emodin sufficiently inhibited the phosphorylation of AKT and its downstream signaling cascades, such as 4E-BP1, p70S6K, and ribosomal protein S6. In addition, the decrease in phosphorylation of AKT by emodin treatment was partly recovered upon supplementation with cholesterol (Figure 6C), which suggested that cholesterol depletion mediated the emodin-induced suppression of AKT signaling.

### 2.7. Cholesterol-Lowering Effects of Emodin Caused STAT3 Phosphorylation and Associated Expression of Cell Cycle Regulating Genes in HCC Cells

**Figure 7:**
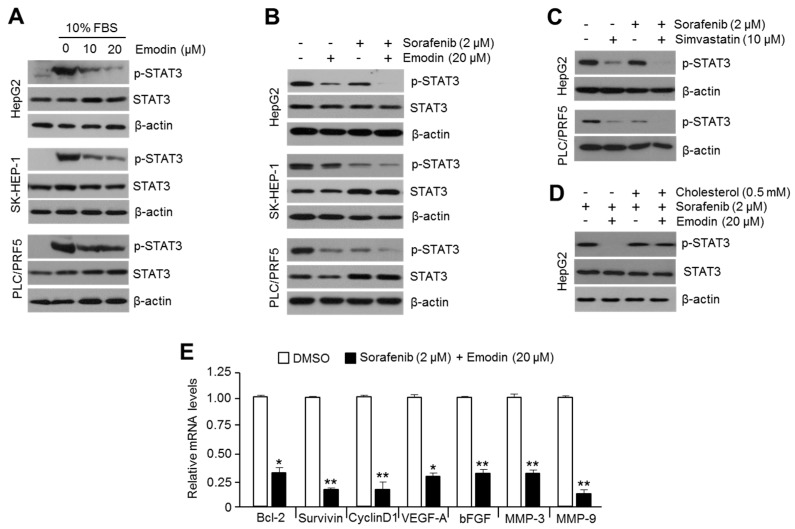
The combination treatment of emodin and sorafenib synergistically suppressed STAT3 and the associated expression of cell cycle-regulating target genes. (**A**) Emodin inhibits the phosphorylation of STAT3. Three HCC cell lines were incubated with emodin (10 and 20 μM) for 24 h. (**B**) The synergistic effect of the emodin and sorafenib combination on STAT3 phosphorylation. The cells were incubated with 20 μM of emodin, 2 μM of sorafenib, or their combination (20 μM emodin and 2 μM sorafenib) for 24 h. (**C**) Simvastatin increased the suppressive effect of sorafenib on STAT3 phosphorylation. HepG2 and PLC/PRF5 cells were incubated with 2 μM of sorafenib, 10 μM of simvastatin, or the combination of sorafenib and simvastatin, as indicated, for 24 h. (**D**) The supplementation of cholesterol blocked the downregulation of STAT3 phosphorylation after the combination treatment of emodin and sorafenib. HepG2 cells were treated with water-soluble cholesterol (0.5 mM) for 1 h prior to the combination treatment of emodin and sorafenib, and the cells were incubated for a further 24 h. Protein expression was measured by Western blotting. (**E**) The combination of emodin and sorafenib decreased the expression of cell cycle-promoting STAT3 target genes. HepG2 cells were incubated for 24 h with the combination (20 μM emodin and 2 μM sorafenib). The values represent the mean ± SD of three independent experiments performed in duplicate; * *p* < 0.05 and ** *p* < 0.01.

AKT-mediated oncogenic growth signaling supports tumor growth via the activation of various transcription factors that regulate the gene expression related to cell cycle progression and anti-apoptotic properties [25]. Initially, we identified that emodin strongly decreased phosphorylated STAT3 in HepG2, SK-HEP-1, and PLC/PRF5 cells (Figure 7A); moreover, the synergistic suppression of STAT3 after the combination of emodin and sorafenib treatment was observed in several HCC cells (Figure 7B). To validate whether the synergistic effects of emodin on the suppression of phosphorylated STAT3 was dependent on decreased levels of intracellular cholesterol, the combinatory effect of simvastatin and sorafenib on STAT3 phosphorylation was analyzed. Similarly, the phosphorylation of STAT3 was markedly decreased by the combination of simvastatin and sorafenib compared with the single drug treatment in HepG2 and PLC/PRF5 cells (Figure 7C). As shown in Figure 7D, cholesterol supplementation partly rescued the decreased STAT3 phosphorylation induced by emodin and sorafenib in HepG2 cells. As the phosphorylation of STAT3 leads to the transcriptional activation of gene expression related to cancer aggressiveness [26], the alteration of STAT3 target genes after the combination treatment of emodin and sorafenib was measured. Consequently, large decreases in the expression of STAT3 target genes, such as B-cell lymphoma 2 (BCL-2), survivin, cyclin D1, vascular endothelial growth factor-A (VEGF-A), basic fibroblast growth factor (bFGF), matrix metalloproteinase-3 (MMP-3), and matrix metalloproteinase-9 (MMP-9), were observed in both emodin and sorafenib-treated HepG2 cells (Figure 7E). These results suggested that the combination of emodin and sorafenib synergistically suppressed the phosphorylation of STAT3 through a cholesterol-lowering effect.

### 2.8. Combination Therapy of Emodin and Sorafenib Suppressed Tumor Growth In Vivo

**Figure 8:**
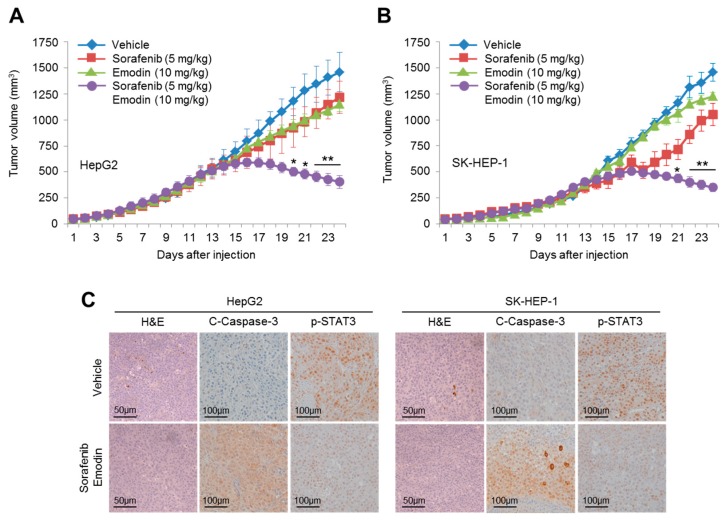
Emodin synergistically increased the anti-cancer efficacy of sorafenib in vivo. (**A**) HepG2 and (**B**) SK-HEP-1 cells were injected into the flanks of nude mice, and the mice were then divided into four groups. When the tumor volumes reached 250 mm^3^, the mice were treated with DMSO, emodin (10 mg/kg/day), sorafenib (5 mg/kg/day), or the combination (10 mg/kg/day emodin and 5 mg/kg/day sorafenib) for three weeks; with the tumor volumes measured daily. Tumor growth curves are plotted as the mean ± SEM (*n* = 7); * *p* < 0.05 and ** *p* < 0.01. (**C**) The combination of emodin and sorafenib decreased the phosphorylation of STAT3 and increased cleaved-caspase-3. The protein expression in xenografted tumor tissues was measured by immunohistochemistry using the indicated antibodies.

The synergistic anti-cancer effect of the combination of emodin and sorafenib was further validated in xenografted HepG2 and SK-HEP-1 mice. Tumor volumes were markedly decreased by the combination treatment of emodin and sorafenib compared with the single treatment with emodin or sorafenib in HepG2 (Figure 8A) and SK-HEP-1 (Figure 8B) tumors. In addition, increased cleaved-caspase-3, an apoptotic marker, and decreased phospho-STAT3 were observed in HepG2 (left panel) and SK-HEP-1 (right panel)-driven tumor tissues treated by the combination of emodin and sorafenib (Figure 8C). These results suggested that emodin synergistically enhanced the anti-cancer efficacy of sorafenib in vivo.

## 3. Discussion

The anti-cancer effect of emodin has been reported in several types of cancer; however, the development of novel therapeutic strategies for cancer treatment is required [27]. In particular, various reports have shown that emodin decreases HCC growth through the suppression of oncogenic growth signaling, such as the MAPK, PI3K-AKT, and STAT3 pathways, in both in vitro and in vivo models [9,28,29]. Nevertheless, the molecular mechanism by which emodin suppresses multiple oncogenic signaling pathways is poorly understood. In the present study, we demonstrated that emodin suppressed PI3K-AKT and STAT3 signaling through its cholesterol-lowering effect.

Cholesterol, as an essential component for lipid raft microdomains in the cellular membrane, is important for cancer growth and aggressiveness and for maintaining oncogenic growth signaling through the PI3K-AKT and STAT3 pathways [20,30,31,32,33]. Experimental or clinical evidence has previously shown that high levels of blood cholesterol accelerate hepatocarcinogenesis and lead to poor clinical outcomes in patients with HCC [30,31]. Indeed, the anti-cancer and anti-metastatic effects of cholesterol-lowering drugs, such as simvastatin, lovastatin, and pitavastatin, which are mediated through suppression of the AKT and STAT3 pathways, have been shown in HCC, renal cancer, malignant melanoma, and ovarian cancer [22,23,24,32,33,34]. Our results show that emodin has a cholesterol-lowering effect through the suppression of SREBP-2 transcriptional activity and its cholesterogenic gene expression in HCC cells. Interestingly, it was demonstrated that fluvastatin, a member of the statin family, was able to sensitize the anti-cancer efficacy of sorafenib in HCC cells, which suggested that cholesterol-lowering drugs may be useful for the improvement of clinical outcomes in patients with advanced HCC who require sorafenib treatment [35]. Similarly, we found that the supplementation of water-soluble cholesterol significantly blocked the decrease in cell viability, as well as AKT and STAT3 signaling, upon the combination therapy of emodin and sorafenib. Therefore, we have proposed that the critical mechanism through which emodin sensitizes HCC cells to the anti-cancer effects of sorafenib is the suppression of AKT and STAT3 oncogenic growth signaling caused by decreases in intracellular cholesterol.

Although sorafenib is a molecular targeted therapy used for the standard treatment in patients with advanced HCC, no significant differences in overall survival have been reported [1,3]. To overcome the lower efficacy of sorafenib in the treatment of advanced HCC, different types of small molecules were tested for their suitability as a combination therapy with sorafenib. For example, Chen et al. [36] reported that 0.5 mg/kg bortezomib synergistically suppressed xenografted PLC/PRF/5 tumors when treated in combination with 5 mg/kg sorafenib by approximately 50% compared with 5 mg/kg sorafenib treatment alone. The sensitizing efficacy of rapamycin (1 mg/kg) in combination with 50 mg/kg sorafenib on the suppression of xenografted patient-derived HCC has also been observed [37]. In addition, a selective MEK inhibitor, AZD6244 (25 mg/kg), showed approximately 30–40% greater anti-cancer efficacy with sorafenib (25 mg/kg) compared with the sorafenib-treated group in patient-derived xenograft models [38]. These previous results suggest that bortezomib, rapamycin, and AZD6244 could be used to improve sorafenib-based treatment in advanced HCC. It has become clear that rapamycin-based therapy causes many side effects, including thrombocytopenia, hyperlipidemia, impaired wound healing, nephrotoxicity, and altered insulin sensitivity [39]. Indeed, serious side effects, such as anemia (9%), hyperglycemia (10%), lymphopenia (14%), oral mucositis (11%), and thrombocytopenia (11%), have been observed in clinical trials with the concurrent therapy of insulin-like growth factor-1 receptor (IGF-1R) inhibitor, cixutumumab, and temsirolimus (a derivative of rapamycin) in patients with bone or soft-tissue sarcoma [40]. Several side effects associated with bortezomib, such as fatigue, diarrhea, vomiting, hematological toxicity, and peripheral neuropathy, have also been reported [41]. In addition, dermatological side effects, such as papulopustular rash, xerosis, pruritus, hyperpigmentation, and weakness to bacterial infection, were associated with AZD6244 [42]. However, it was previously reported that 25–40 mg/kg emodin, a higher dosage than that used in our study, significantly reduced tumor growth in colorectal, prostate, myelocytic leukemia, and HCC-derived xenografted tumors, with no adverse side effects observed [29,43,44,45]. In the present study, we have shown that the combination treatment of emodin (10 mg/kg) and sorafenib (5 mg/kg), which was lower than previously studied combinations, more effectively reduced tumor growth, by approximately 50–70% more than the single treatment of sorafenib in HepG2 or SK-HEP-1-transplanted xenograft models, which suggested that emodin could be clinically applicable for the improved therapeutic efficacy of sorafenib in advanced HCC without the induction of side effects.

Collectively, our in vitro and in vivo experimental findings indicated that the anti-cancer effect of emodin was mediated through the suppression of intracellular cholesterol levels, and subsequently caused the attenuation of oncogenic AKT and STAT3 pathways; in addition, we confirmed that this would provide a promising therapeutic strategy for improvement of the anti-cancer efficacy of sorafenib in patients with advanced HCC.

## 4. Materials and Methods

### 4.1. Reagents and Antibodies

Emodin, sorafenib, water-soluble cholesterol, doxorubicin, 5-fluorouracil, and simvastatin were purchased from Sigma Aldrich (St. Louis, MO, USA) and Santa Cruz Biotechnology (Dallas, TX, USA). Antibodies recognizing phospho-4EBP1T70 (sc-18092), 4EBP1 (CST-9452), phospho-p70S6K1 (CST-9234), HMGCS1 (CST-36877), HMGCR (ab174830), FDPS (ab189874), AKT (CST-4691), phospho-AKTS473 (CST-4060), STAT3 (CST-4904), phospho-STAT3 (CST-9145), cleaved-caspase-3 (CST-9664), and β-tubulin (sc-9104) were purchased from Cell Signaling Technology (Danvers, MA, USA), Santa Cruz Biotechnology, and Abcam (Cambridge, MA, USA).

### 4.2. Cell Culture and Cell Viability Assay

HCC cell lines (Hep3B, HepG2, SK-HEP-1, Huh7, PLC/PRF5) were obtained from the Korean Cell Line Bank (Seoul, Korea) and American Type Culture Collection (Manassas, VA, USA) and cultured in Dulbecco’s modified Eagle’s medium (DMEM), Roswell Park Memorial Institute medium (RPMI1640), and Minimum Essential Medium Eagle alpha medium (MEM-α) supplemented with 10% fetal bovine serum. To measure cell viability, the cells were seeded into 12- or 24-well tissue culture dishes and incubated for 24, 48, and 72 h with or without drugs to test the anti-cancer efficacy. After incubation with the test drugs, the cells were washed and fixed with phosphate-buffered saline (PBS) and, subsequently, with 4% paraformaldehyde. The cells were stained with 0.5% crystal violet solution for 20 min at room temperature, the crystal violet-stained cells were solubilized in 1% SDS solution, and the optical density was measured at 570 nm using an absorbance reader (BioTek, Winooski, VT, USA) (OD570).

### 4.3. Western Blotting

For Western blotting, the crude protein samples were extracted from cultured cells using protein extraction buffer (1% IGEPAL, 150 mM NaCl, 50 mM Tris-HCl (pH 7.9), 10 mM NaF, 0.1 mM EDTA, and a protease inhibitor cocktail). SDS-PAGE was applied to separate the protein samples by molecular weight, and the separated proteins were then transferred onto PVDF membranes (Millipore, Burlington, MA, USA). The membranes with separated proteins were incubated with primary antibodies (1:1000–1:5000 dilution) and horseradish peroxidase (HRP)-conjugated secondary antibodies (1:10,000) at 4 °C and room temperature, respectively. The Enhanced Chemiluminescence (ECL) Prime kit (GE Healthcare, Pittsburgh, PA, USA) was used for the analysis of protein expression.

### 4.4. Quantitative Real-Time PCR

Quantitative real-time PCR was performed as previously described [46]. Briefly, 2 μg of total RNA extracted from cultured cells by using TRIzol (Invitrogen, Waltham, MA, USA) and cDNA synthesis was performed by using a high-capacity cDNA reverse transcription kit (Applied Biosystems, Waltham, MA, USA). Quantitative PCR was performed by using SYBR Green PCR Master Mix (Applied Biosystems, Waltham, MA, USA). The primer sequences used in the experiment are shown in Table 1.

### 4.5. Tumor Xenograft Assay and Immunohistochemistry

As previously described [46], BALB/c-nude mice were used as human tumor xenograft models. HepG2 or SK-HEP-1 cells (1 × 10^6^) were harvested and suspended in 100 µL of FBS-free medium. Then, the cells were innoculated into the flank of mice. Upon reaching a tumor size of 250 mm^3^, the mice were intraperitoneally injected with emodin (10 mg/kg), sorafenib (5 mg/kg), a combination of emodin (10 mg/kg) and sorafenib (5 mg/kg), or vehicle every day for three weeks. The tumor size was measured using calipers and calculated from the following equation: volume = ab^2^/2, where a is the maximal width and b is the maximal orthogonal width. To measure the protein expression determined by immunohistochemistry, tumor tissues were fixed with 4% paraformaldehyde, embedded in paraffin, and then divided into sections of 6 μm in thickness. The tissue sections were deparaffinized, rehydrated, and autoclaved for 10 min in 10 mM sodium citrate (pH 6.0) to retrieve the target antigens. These sections were then incubated overnight at 4°C with parimary antibodies such as anti-cleaved-caspase-3 (1:50; CST-9991) and anti-phospho-STAT3 (1:50; CST-9145). The following day, the sections were incubated with biotinylated secondary antibodies, and finally visualyzed with diaminobenzidine (DAB). The animal experiments were conducted and managed in accordance with the guidelines of the Konkuk University Institutional Animal Care and Use Committee (KU16036-1).

### 4.6. Cell Cycle Analysis

We performed cell cycle analysis following the manufacturer’s instructions (Millipore, Burlington, MA, USA). Cells were fixed with 70% ethanol under −20 °C for 3–5 h. Afterwards, the cells were reacted with Muse™ cell cycle assay kit reagent (200 µL) for 30 min at room temperature in the dark. The cell populations were analyzed using a Mini Flow Cytometry Muse™ Cell Analyzer (Millipore, Burlington, MA, USA).

### 4.7. Ki67 Cell Proliferation Assay

The Muse™ Ki67 Proliferation Kit was used to measure cell proliferation in accordance with the supplier’s instructions (Millipore, Burlington, MA, USA). The cells were seeded at a density of 1 × 10^5^ cells/well into a 6-well tissue culture plate and incubated with or without drugs that tested anti-cancer efficacy. After incubation, the cells were washed with PBS and incubated with fixative solution for 15 min at room temperature. To increase the permeability of the cell membrane to the Ki67 antibody, 100 µL of permeabilization solution was reacted with fixed cells for 15 min at room temperature. To stain the Ki67 protein, used as a marker of cell proliferation, 10 µL Muse™ Hu Ki67 antibody was incubated with 50 μL of sample for 30 min at room temperature in the dark. The proliferating cell populations were analyzed using a Mini Flow Cytometry Muse™ Cell Analyzer (Millipore, Burlington, MA, USA).

### 4.8. Apoptosis Assays

Annexin-V staining was used to determine the proportion of apoptotic cells in accordance with a previously described experimental procedure [46]. Briefly, 1 × 10^5^ cells/well were seeded onto a 6-well cell culture plate and incubated with emodin (20 μM), sorafenib (2 μM), or combination of emodin (20 μM) and sorafenib (2 μM) for 72 h. After drug treatment, the cells were collected into fresh tubes, washed with cold PBS, and centrifuged at 2000 rpm for 2 min at room temperature. The cell pellets were incubated in 100 μL Muse™ Annexin V and Dead Cell kit reagents (Millipore, Burlington, MA, USA) for 20 min at room temperature. The Mini Flow Cytometry Muse™ Cell Analyzer (Millipore, Burlington, MA, USA) was applied for the measurement of the apoptotic cell numbers.

### 4.9. Measurement of Intracellular Cholesterol

We measured the total cellular cholesterol using the Amplex Red cholesterol assay kit (Invitrogen, Waltham, MA, USA) in accordance with the manufacturer’s protocols. Briefly, prepared cultured cells were harvested and kept at −70 °C. The frozen cells were incubated with reaction buffer for 30 min on ice, and then sonicated to disrupt the cellular membrane. Samples (50 µL) were mixed with equal volume of Amplex Red reagent containing HRP, cholesterol oxidase, and cholesterol esterase, and then the mixture was reacted for 30 min at 37 °C. The fluorescence intensity was measured using a fluorescence microplate reader (BioTek, Winooski, VT, USA) at ex/em = 530/590. The cholesterol levels were normalized using protein concentration determined by Bradford assay.

### 4.10. Luciferase Assay

The steroid responsive element (SRE)-wild type (WT) or -mutant (Mut) containing luciferase vector (pSynSRE-T-Luc and pSynSRE-Mut-T-Luc) was transfected into SK-HEP-1 cells using Lipofectamine 2000 (Invitrogen, Waltham, MA, USA). pSynSRE-T-Luc (Addgene plasmid #60444) and pSynSRE-Mut-T-Luc (Addgene plasmid #60490) were gifts from Timothy Osborne [47]. Luciferase activities were analyzed using a Synergy 2 Luminometer (BioTek, Winooski, VT, USA) and normalized against the activity of β-galactose.

### 4.11. Statistical Analysis

All statistical analyses were performed using the unpaired Student’s *t*-test and one-way analysis of variance (ANOVA) with Tukey post hoc test; the data are presented as the mean ± standard deviation (SD). For combination studies with emodin and sorafenib in cell-based experiments and mouse xenografts, the two-way ANOVA with Tukey post hoc test was performed, and the data are represented as the mean ± standard error of the mean (SEM) [48]. A *p* value of less than 0.05 was considered statistically significant.

## 5. Conclusions

The major findings of this study are that emodin synergistically increases anticancer efficacy of sorafenib through suppression of oncogenic growth signaling and STAT3-mediated cell cycle progression and proliferation by attenuating cholesterol biosynthesis in vitro and in vivo. Overall, these results suggested that the combination of emodin and sorafenib may be potentially therapeutic for patients with advanced HCC.

## Figures and Tables

**Table 1:** Primer sequences for quantitative real-time PCR.

Gene	Forward Primer	Reverse Primer
***HMGCS1***	TGGCAGGGAGTCTTGGTA	TCCCACTCCAAATGATGACA
***HMGCR***	GATGGGAGGCCACAAAGAG	TTCGGTGGCCTCTAGTGAGA
***MVD***	TTAACTGGTCCTGGTGCAGA	AACATCGCGGTCATCAAGTA
***FDPS***	TCCATGATGTCATCTGCCAC	AGCCAAGGAAACAGGATG
***DHCR7***	CTTGAGATGCGGTTCTGTCA	TATTTGGCAAGAGGCTGGAG
***DHCR24***	CTGAAGACAAACCGAGAGGG	TGTTGCCAAAGGGGATAATG
***BCL-2***	CGTACAGTTCCACAAAGGCA	ATGTGTGTGGAGAGCGTCAA
***Survivin***	CTTTCTCCGCAGTTTCCTCA	TTGGTGAATTTTTGAAACTGGA
***Cyclin D1***	ATGGAACACCAGCTCCTGTGCTGC	TCAGATGTCCACGTCCCGCACGT
***VEGFA***	AGCTGCGCTGATAGACATCC	CTACCTCCACCATGCCAAGT
***bFGF***	CCGACGGCCGAGTTGAC	TAACGGTTAGCACACACTCCTTTG
***MMP-3***	ACAAAGGATACAACAGGGACCA	GTGAGTGAGTGATAGAGTGGGT
***MMP-9***	CAGTCCACCCTTGTGCTCTT	CCCGAGTGTAACCATAGCGG

## Abbreviations

HCC	Hepatocellular carcinoma
SREBP-2	Sterol regulatory element-binding protein-2
AKT	Protein kinase B
STAT3	Signal transducer and activator of transcription 3
MAPK	Mitogen-activated protein kinase
PI3K	Phosphoinositide 3-kinase
HMGCS1	3-hydroxy-3-methylglutaryl-CoA synthase 1
HMGCR	3-hydroxy-3-methylglutaryl-coenzyme A reductase
FDPS	Farnesyl diphosphate synthase
MVD	Mevalonate diphosphate decarboxylase
DHCR7	7-dehydrocholesterol reductase
DHCR24	24-dehydrocholesterol reductase
5-FU	5-fluorouracil
BCL-2	B-cell lymphoma 2
VEGF-A	Vascular endothelial growth factor A
bFGF	Basic fibroblast growth factor
HGF	Hepatocyte growth factor
MMP-3	Matrix metalloproteinase-3
MMP-9	Matrix metalloproteinase-9
